# Key Role of the Endothelial TGF-β/ALK1/Endoglin Signaling Pathway in Humans and Rodents Pulmonary Hypertension

**DOI:** 10.1371/journal.pone.0100310

**Published:** 2014-06-23

**Authors:** Benoît Gore, Mohamed Izikki, Olaf Mercier, Laurence Dewachter, Elie Fadel, Marc Humbert, Philippe Dartevelle, Gerald Simonneau, Robert Naeije, Franck Lebrin, Saadia Eddahibi

**Affiliations:** 1 INSERM U999, Le Plessis-Robinson, France; 2 Laboratory of Physiology, Faculty of Medicine, Free University of Brussels, Brussels, Belgium; 3 Centre Chirurgical Marie Lannelongue, Le Plessis-Robinson, France; 4 INSERM U833, Collège de France, Paris, France; University of Giessen Lung Center, Germany

## Abstract

Mutations affecting transforming growth factor-beta (TGF-β) superfamily receptors, activin receptor-like kinase (ALK)-1, and endoglin (ENG) occur in patients with pulmonary arterial hypertension (PAH). To determine whether the TGF-β/ALK1/ENG pathway was involved in PAH, we investigated pulmonary TGF-β, ALK1, ALK5, and ENG expressions in human lung tissue and cultured pulmonary-artery smooth-muscle-cells (PA-SMCs) and pulmonary endothelial cells (PECs) from 14 patients with idiopathic PAH (iPAH) and 15 controls. Seeing that ENG was highly expressed in PEC, we assessed the effects of TGF-β on Smad1/5/8 and Smad2/3 activation and on growth factor production by the cells. Finally, we studied the consequence of ENG deficiency on the chronic hypoxic-PH development by measuring right ventricular (RV) systolic pressure (RVSP), RV hypertrophy, and pulmonary arteriolar remodeling in ENG-deficient (*Eng^+/−^*) and wild-type (*Eng^+/+^*) mice. We also evaluated the pulmonary blood vessel density, macrophage infiltration, and cytokine expression in the lungs of the animals. Compared to controls, iPAH patients had higher serum and pulmonary TGF-β levels and increased ALK1 and ENG expressions in lung tissue, predominantly in PECs. Incubation of the cells with TGF-β led to Smad1/5/8 phosphorylation and to a production of FGF2, PDGFb and endothelin-inducing PA-SMC growth. Endoglin deficiency protected mice from hypoxic PH. As compared to wild-type, *Eng^+/−^* mice had a lower pulmonary vessel density, and no change in macrophage infiltration after exposure to chronic hypoxia despite the higher pulmonary expressions of interleukin-6 and monocyte chemoattractant protein-1. The TGF-β/ALK1/ENG signaling pathway plays a key role in iPAH and experimental hypoxic PH via a direct effect on PECs leading to production of growth factors and inflammatory cytokines involved in the pathogenesis of PAH.

## Introduction

Pulmonary arterial hypertension (PAH) is an uncommon but potentially life-threatening disease. Incompletely understood pathogenic mechanisms cause a progressive increase in pulmonary vascular resistance that ultimately leads to right ventricular (RV) failure [1]. Among patients with heritable forms of PAH, many have germline mutations in genes encoding receptors of the transforming growth factor-beta (TGF-β) receptor superfamily, usually the bone morphogenetic protein (BMP) receptor type 2 gene (*BMPR2*) and less often the *ACVRL1* or *ENG* gene encoding activin receptor-like kinase 1 (ALK1) and endoglin (ENG), respectively. These germline mutations have also been linked to hereditary hemorrhagic telangiectasia (HHT), an autosomal dominant vascular disorder characterized by telangiectasia and arteriovenous malformations [2].

TGF-β is a multifunctional cytokine whose signals are mediated by specific type I and II serine/threonine kinase receptors. Upon binding of active TGF-β to the constitutively activated type II receptor (TβRII), a specific type I receptor (TβRI) is recruited. Activation of this second receptor via phosphorylation initiates intracellular signaling through the phosphorylation of a set of receptor-regulated Smads (R-Smad), which subsequently form a complex with co-Smad (Smad4). The R-Smad/co-Smad complex enters the nucleus, where it modulates the transcription of target genes. In most cells, the TGF-β signaling pathway involves the TβRII/ALK5 complex, which induces Smad2/3 phosphorylation. However, in endothelial cells, TGF-β activates two distinct type I receptors, ALK5 and ALK1, which transmit signals via the ALK5/Smad2/3 and ALK1/Smad1/5 pathways, respectively [3]. ALK5/Smad2/3 inhibits and ALK1/Smad1/5 stimulates endothelial-cell proliferation and migration. ENG, which is a transmembrane accessory receptor for TGF-β signaling, plays a pivotal role in the balance of ALK1 and ALK5 signaling that regulates endothelial cell proliferation. ENG is predominantly expressed on proliferating endothelial cells *in vitro* and on angiogenic blood vessels *in vivo* [4].The TGF-β/ALK1/ENG signaling pathway plays a key role in vessel formation and maintenance [4]. However, its contribution to the pathogenesis of PAH is poorly understood [4]. In the present study, we evaluated the susceptibility of ENG-deficient mice (*Eng^+/−^*) to PAH induced by 3 weeks of chronic hypoxia. We also evaluated pulmonary vascular remodeling and inflammation in these mice comparatively with wild-type mice exposed to chronic hypoxia. We investigated the expression pattern of the TGF-β/ALK1/ENG signaling pathway in lung tissue and in pulmonary-artery smooth-muscle-cells (PA-SMCs) and pulmonary endothelial cells (PECs) from patients with iPAH comparatively with controls. In a recent study, we found that serum-free medium of quiescent human PECs elicited marked PA-SMC proliferation [5]. We therefore evaluated whether TGF-β exposure of cultured PECs from controls increased the growth-promoting activity of the PEC culture medium and modified the expression of growth-promoting factors implicated in PAH development.

## Methods

### Ethics Statement

This study was approved by the institutional review board and the local ethics committee (Comité de Protection des Personnes, Ile-de-France VII, Le Kremlin-Bicêtre, France). Written, informed consent was given by all the patients prior to their contribution to the study.

Experiments were conducted according to the European Union regulations (Directive 86/609 EEC) for animal experiments and complied with our institution's guidelines for animal care and handling. The animal facility is licensed by the French Ministry of Agriculture (agreement N° B92-019-01). This study was approved by the Committee on the Ethics of Animal Experiments CEEA26 CAPSud. All animal experiments were supervised by Dr. Olaf Mercier (agreement delivered by the French Ministry of Agriculture for animal experiment N° A92–396). All efforts were made to minimize animal suffering.

### Studies of human lung tissue samples

#### Tissue sampling

Lung tissue and pulmonary arteries were collected during lung transplantation in 14 patients (8 men and 6 women) with idiopathic pulmonary hypertension (iPAH) and during lobectomy or pneumonectomy for localized cancer in 15 controls (9 men and 6 women). All iPAH patients had New York Heart Association class III or IV heart failure and were treated with intravenous epoprostenol. In the controls, preoperative transthoracic echocardiography showed no evidence of PAH and the study samples were taken at a distance from tumor sites. Neither the iPAH patients nor the controls had *BMPR2*, *ACVRL1*, or *ENG* mutations or polymorphisms.

In the patients with iPAH, mean pulmonary artery pressure was 61±4 mm Hg (range, 28 to 90 mm Hg), mean pulmonary vascular resistance was 12.53±1.30 mm Hg ·L*^−^* ^1^·min*^−^* ^1^· m*^−^* ^2^ (range, 5.67 to 22.40 mm Hg·L*^−^* ^1^·min*^−^* ^1^·m*^−^* ^2^), and mean cardiac index was 2.42± 0.2 L·min*^−^* ^1^·m*^−^* ^2^ (range, 1.2 to 3.4 L·min*^−^* ^1^·m*^−^* ^2^).

#### Isolation and culture of human PA-SMCs and PECs

Human PA-SMCs were cultured from pulmonary artery explants, as previously described [6]. To characterize the PA-SMC phenotype, we assessed the expression of muscle-specific contractile and cytoskeletal proteins including smooth-muscle-cell α-actin, desmin, and vinculin [6]. PA-SMCs were used between passages 3 and 6.

Human PECs were obtained by exposing lung tissue fragments to Dispase I digestion (Roche Diagnostics, Penzbeg, Germany) at 37°C overnight, as previously described [5]. The suspension was filtered, plated onto 0.1% gelatin-coated wells, and grown in MCDB131 medium (Invitrogen, Cergy-Pontoise, France) supplemented with 10% fetal calf serum (FCS), 50 U/mL penicillin/streptomycin, 4 mmol/L L-glutamine, 25 mmol/L HEPES, 10 U/mL heparin, 1 µg/mL human endothelial cell growth supplement, and 10 ng/mL vascular endothelial growth factor (Promocell, Heidelberg, Germany). PECs were purified using the immunomagnetic technique with anti-PECAM-1 (CD31) monoclonal antibody-labeled DynaBeads14 (Dynal, Biotech, Compiègne, France). For endothelial-cell phenotype characterization, the cells were labeled with acetylated low-density lipoprotein (LDL) coupled to a fluorescent carbocyanine dye (DiI-Ac-LDL, Tebu, Le Perray en Yvelines, France) and stained with antibodies against the endothelial cell-specific lectin *Ulex europaeus* agglutinin-1 (UEA-1, Sigma, Lyon, France). Experiments were also performed using monoclonal antibodies against desmin and vimentin (Dako, Trappes, France). Cells positive for DiI-Ac-LDL and UEA-1 and negative for desmin and vimentin were classified as endothelial cells and constituted >95% of our PEC cultures. Cells were used for the study at passage 6 [5].

#### Protein extraction and Western blotting

Lung samples were homogenized, and PECs and PA-SMCs were sonicated in lysis CHAPS Buffer (150 mM NaCl, 10 mM Tris HCl pH = 7.5, 1 mM EDTA, 1 mM EGTA, 1 mM Leupeptin, 1 mM PMSF, and 1% CHAPS). Protein concentrations were measured using the Bradford technique, and 30 µg of protein per sample was used for Western blotting (10% acrylamide), as previously described [5]. Proteins were electrophoretically transferred to a nitrocellulose membrane (Sigma-Aldrich, Ayrshire, UK), which was then saturated in 5% bovine serum albumin (BSA) in Tris-buffered saline Tween 20 (TBS-T). TGF-β receptor protein expression was evaluated using goat anti-human ALK1, ALK5, and ENG antibodies (R&D System, Lille), diluted 1∶1000 in BSA with 1% TBS-T. Detection was with an anti-goat antibody coupled to horseradish peroxidase (HRP) (Dakocytomation, Trappes, France) diluted 1∶2000 in BSA, 1% TBS-T, and ECL kit substrate (GE Healthcare, Vélizy, France). A polyclonal antibody against β-actin (diluted 1∶3000; Sigma Aldrich) served as the internal control. Densitometric quantification of the immunoblot bands was performed using Bio-Rad Quantity One software.

#### Enzyme-linked immunosorbent assay (ELISA)

TGF-β protein levels were evaluated in lung samples and serum from patients with iPAH and controls using an ELISA (R&D systems, Lille, France). ENG protein concentrations were determined in the same serum samples and in supernatants from PECs isolated from patients with iPAH and controls using ELISA kits, according to the manufacturer's instructions (R&D Systems, Lille).

#### Effect of TGF-β on Smad phosphorylation

PECs (300 000 cells per well, 6-well plates coated with 0.1% gelatin) were plated in 10% FCS/MCB131 medium for 24 hours then starved for 24 hours in serum-free medium. PECs were then treated with TGF-β (0, 5, or 50 ng/mL) for 30 min. The protein was harvested by scraping in a cell lysis buffer containing an anti-phosphatase mixture (Cell Signaling, Saint-Quentin, France) then sonicated. Protein concentrations were measured using the Bradford technique, and 10 µg samples were then used for Western blotting as previously described [5]. Immunoblotting assays were performed as described above with rabbit anti-human phospho-Smad1/5/8 (Cell Signaling, Saint-Quentin, France) and rabbit anti-human Smad 1/5/8 (Santa Cruz Biotechnology, Le Perray en Yvelines, France) diluted 1∶1000 to determine the ratio of phospho-Smad1/5/8 over Smad1/5/8. Detection was performed with anti-rabbit antibody coupled to HRP (Dakocytomation, Trappes, France).

#### RNA extraction

Total RNA was extracted from lung tissue, PECs, and PA-SMCs using RNeasy Mini kit (Qiagen SA, Courtaboeuf, France), according to the manufacturer's instructions. RNA integrity was evaluated by visual inspection of ethidium bromide-stained agarose gels and the RNA concentration was determined from optical density measurements.

#### Preparation of cDNA and quantitative real-time polymerase chain reaction (QRT-PCR)

First-strand cDNA synthesis was with the SuperScript II RT reverse transcriptase system (Invitrogen, Cergy Pontoise). A mix containing 1 µg of total RNA, 100 ng of random primers, and 1 µL of dNTP (10 mM) in a total of 12 µL was incubated for 5 min at 65°C and chilled on ice. After addition of a second mixture of 4 µL of first-strand buffer, 2 µL of dithiothreitol (0.1 M), and 40 U of RNase inhibitor (RNaseOUT; Invitrogen, Cergy Pontoise), the samples were left at 25°C for 2 min. Finally, after addition of 1 µL of SuperScript II RT (200 units/µL), the reaction was incubated for 10 min at 25°C, 50 min at 42°C, and 15 min at 70°C. The cDNA was diluted 1∶20 for use in a quantitative real-time-polymerase chain reaction (QRT-PCR).

The primers were designed using Primer Express Software (Applied Biosystems, Courtabœuf, France) for human *TGF-β*, *ALK1*, *ALK5*, and *ENG* and for both human and mouse preproendothelin-1 (preproET-1), platelet-derived growth factor (PDGF)-A and -B, fibroblast growth factor (FGF)-2, epidermal growth factor (EGF), interleukin (IL)-6, and monocyte chemoattractant protein (MCP)-1. Specific QRT-PCR primer sequences for all genes were listed in Table S1. To avoid inappropriate amplification of residual genomic DNA, intron-spanning primers were selected and internal control 18S rRNA primers were used. For each sample, the amplification reaction was performed in duplicate using Syber Green mix and specific primers. Signals were detected and results analyzed using ABI-Prism 7000 sequence detection software (Applied Biosystems). The expression level of the genes of interest was computed relative to the mRNA expression level of the internal standard r18S as follows: relative mRNA = 1/2^(Ctgene of interest-Ctr 18S)^.

#### PA-SMC growth assays

PA-SMCs from controls were seeded at a density of 5·10^4^ cells/per well in Dulbecco's modified Eagle's medium supplemented with 10% FCS and allowed to adhere. The cells were subjected to 48 h of growth arrest in medium containing 0% FCS then treated with 1 mL of the various conditioned media collected from control PECs. PEC serum-free medium was obtained as follows: at the time of initiating PA-SMC growth arrest, PECs were seeded in 24-well plates at a density of 5·10^4^ cells per well and were allowed to adhere and to grow in supplemented MCDB 131 as described above, for 24 h. The PECs were then subjected to 24 h of growth arrest in MCDB 131 medium with 0.2% FCS, with or without 5 ng/mL of TGF-β in the presence or absence of anti-ENG antibody. The medium was removed and used for PA-SMC incubation for 48 h, after which the PA-SMCs were counted.

#### Effect of TGF-β on cytokine induction in PECs

PECs were seeded and synchronized as previously described. The cells were either untreated or treated for 4 h with 5 ng/mL of TGF-β in the presence or absence of anti-ENG antibody. The PECs were then used for QRT-PCR.

### Studies in mice

#### Chronic hypoxia model

ENG-deficient (*Eng^+/−^*)[7] and wild-type (*Eng^+/+^*) transgenic C57BL6 mice were exposed to chronic hypoxia (10% O_2_) in a ventilated chamber (500-L volume, Flufrance) or to normoxia for 3 weeks. The hypoxic environment was established by flushing the chamber with a mixture of room air and nitrogen (10% oxygen, 90% diazote (N_2_) and recirculating the gas. Carbon dioxide was removed using soda lime granules, and excess humidity was prevented by cooling the recirculation circuit. The chamber environment was monitored using an oxygen analyzer.

#### Assessment of pulmonary hypertension in mice

Mice were anesthetized with intraperitoneal sodium pentobarbital (4 mg/100 g). After incision of the abdomen, a pressure transducer was inserted into the RV and RV systolic pressure (RVSP) was recorded, as previously described [8]. To assess RV hypertrophy, the RV was dissected from the left ventricle and septum, and the ratio of RV weight over the weight of the left ventricle and septum (LV+S) was computed (Fulton's index).

The lungs were removed, fixed, and processed for paraffin embedding. The percentage of muscularized vessels was calculated as previously described [8]. Briefly, in each mouse, 60 intra-acinar arteries at a size lower than 80 µm were categorized as non-muscularized (NM), partially muscularized (PM) or fully muscularized (M) to assess the degree of muscularization. The lungs were also removed and stored at −80°C for QRT-PCR.

#### Immunohistochemistry

Paraffin sections (5 µm) of lung specimens were mounted on Superfrost Plus slides (Fisher Scientific, Illkirch, France). The slides were dewaxed in 100% toluene, and the sections were progressively rehydrated by immersion in decreasing ethanol concentrations (100%, 95%, and 70%) and, finally, in distilled water. Endogenous peroxidase activity was blocked with H_2_O_2_ in methanol (0.3% v/v) for 10 minutes. After three washes with phosphate buffer saline (PBS), the sections were preincubated in PBS supplemented with 3% (w/v) BSA for 30 min then incubated overnight at 4°C with antibodies. Mouse PECs were stained with goat polyclonal anti-PECAM-1 antibody (Santa Cruz) diluted 1∶200 and macrophages were stained with rat anti-mouse F4/80 (AbD Serotec, Düsseldorf, Germany) diluted 1∶400. The sections were then exposed for 30 min to biotin-labeled anti-goat secondary antibodies (Dako, Trappes, France) diluted 1∶200 in the same buffer then to streptavidin-biotin horseradish peroxidase solution. For F4/80 staining, the sections were exposed to anti-rat HRP (Santa Cruz) diluted 1∶200 in PBS. Peroxidase staining was carried out using 3,3′-diaminobenzidine tetrahydrochloride dihydrate (DAB, Sigma) and hydrogen peroxide. Finally, the sections were stained with hematoxylin and eosin. Vascular density and macrophage infiltration were assessed in 10 selected fields and stored as digital field images. The results were expressed as vessel number and macrophage number per 100 alveoli.

### Statistical analysis

All data are reported as mean±SEM. Statistical significance was tested using ANOVA or the nonparametric Mann-Whitney test. *P* values <0.05 were considered statistically significant.

## Results

### Pulmonary and cell expression of TGF-β/TGF-β receptors in patients with iPAH and controls

**Figure 1:**
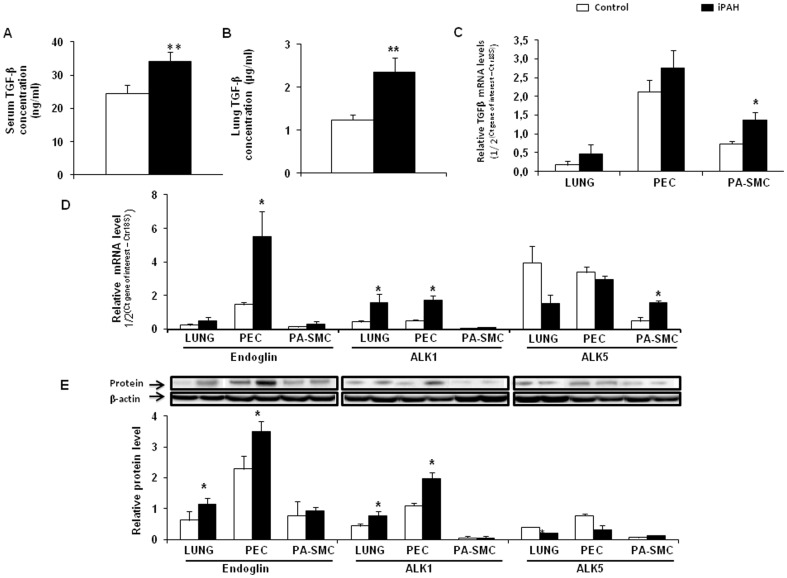
The open bars indicate the results in controls and the closed bars in patients with idiopathic pulmonary hypertension (iPAH). TGF-β was assayed using an ELISA in serum and lung homogenates (controls, n = 15; iPAH patients, n = 14) (**A** and **B**). (**C**) TGF-β mRNA measured in pulmonary endothelial cells (PECs) and pulmonary-artery smooth-muscle cells (PA-SMCs; controls, n = 7; iPAH patients, n = 7). (**D**) TGF-β receptor expression: *ALK1, ALK5*, and *ENG* mRNA measured in PECs and PA-SMCs (controls, n = 7; iPAH patients, n = 7). (**E**): ENG, ALK1, and ALK5 protein expression in LUNG, PECs and PA-SMCs. Protein levels were normalized for β-actin (controls, n = 7; iPAH patients, n = 7). Values are mean±SEM. *P<0.05 and **P<0.01 compared with controls.

TGF-β protein levels were increased in serum and lung tissue homogenates from patients with iPAH, compared to controls (Figure 1A and B). TGF-β mRNA levels were also higher in cultured PA-SMCs from iPAH patients compared to those from controls (Figure 1C).

Compared to controls, lung tissue and PECs from iPAH patients had higher levels of ENG and ALK1 mRNA and proteins (Figure 1D, E, F). ENG protein was also increased in serum and PEC supernatants from patients with iPAH (data not shown). In contrast, in PA-SMCs from both groups, ENG expression was very low and ALK1 was not detected (Figure 1D, and E). ALK5 MRNA levels were increased in iPAH PA-SMCs, but we did not detect any increase of ALK5 protein levels in the lungs, PEC and PA-SMC from patients with iPAH (Figure 1D, and E).

### Differential effects of TGF-β on the Smad signaling pathway in human PECs

**Figure 2:**
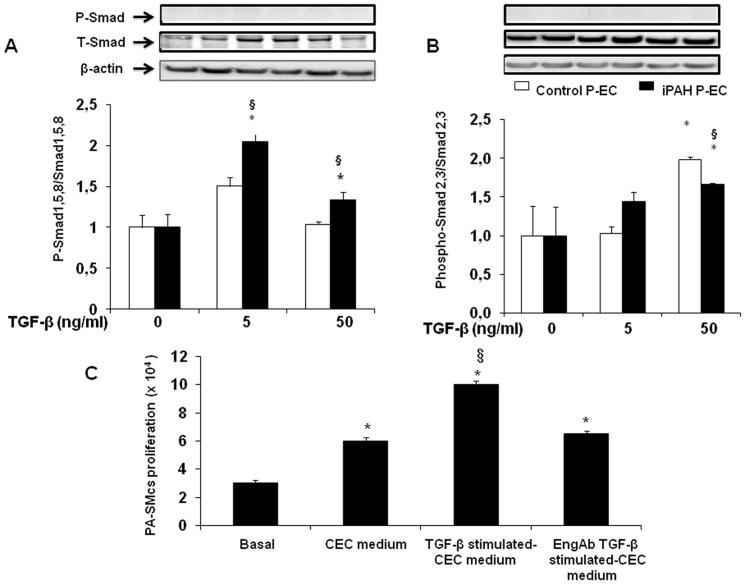
Effect of increasing TGF-β doses on Smad 1,5,8 and Smad 2,3 phosphorylation, respectively, in pulmonary endothelial cells (PECs) from controls and from patients with idiopathic pulmonary hypertension (iPAH) (**A** and **B**). Protein levels were normalized for β-actin. Values are means±SEM normalized for results without TGF-β. *P<0.05 compared to relevant controls without TGF-β, ^§^ *P*<0.05 compared to control PECs under the same conditions. (**C**) Growth of pulmonary-artery smooth-muscle cells (PA-SMCs) from controls in response to serum-free media derived from cultured PECs from controls and stimulated by TGF-β with or without anti-ENG antibody (ENG Ab).Values are mean±SEM. *P<0.05 compared to basal condition, ^§^ *P*<0.05 compared to PA-SMCs stimulated with PEC medium.

Because PECs have been shown to express highly ALK1 and ENG and moderatelyALK5, we evaluated the effects of TGF-β treatment on Smad1/5/8 and Smad2/3 phosphorylation in these cells. Increasing concentrations of TGF-β (0, 5, and 50 ng/mL) induced phosphorylation of Smad1/5/8 in PECs from patients with iPAH and, to a lesser extent, from controls. The maximal effect was observed at 5 ng/mL of TGF-β (Figure 2A). Phosphorylation of Smad2/3 was not detected at this concentration, but a weak signal was observed at 50 ng/mL of TGF-β and remained similar in the two groups (Figure 2B).

### PA-SMC growth in response to medium from cultured PECs treated with TGF-β

Serum-free medium from cultured PECs from controls was added to PA-SMCs from the same individuals cultured without serum. This produced a marked increase in PA-SMC proliferation compared to the basal condition. When the PECs were treated with 5 ng/mL of TGF-β, the conditioned medium had a stronger effect on PA-SMCs. This effect was completely inhibited when TGF-β was combined with anti-ENG antibody (Figure 2C). TGF-β 5 ng/mL added directly to PA-SMCs had no effect (data not shown).

### Effect of TGF-β on PEC paracrine factors influencing PA-SMC growth

**Figure 3:**
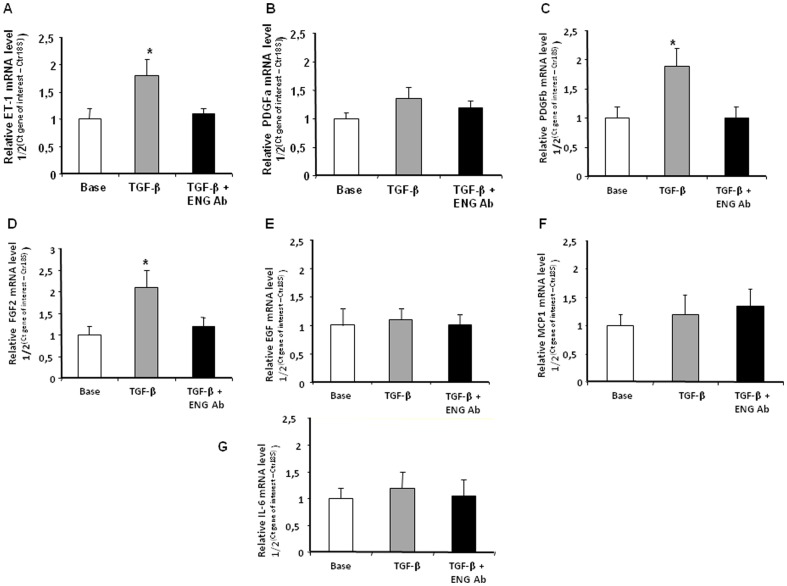
Effect of pulmonary endothelial cells (PECs) from controls incubated with TGF-β with or without anti-ENG antibody (ENG Ab) on mRNA expression of A) *preproET-1*, (B) *PDGFa*, (C) *PDGFb*, (D) *FGF2*, (E) *EGF*, (F) *MCP-1*, and (G) *IL-6*. Values are mean±SEM.*P<0.05 compared to PECs without TGF-β treatment.

Compared to the basal condition, control PECs exposed to TGF-β 5 ng/mL induced a marked increase in the expression of *ET-1, PDGFb*, and *FGF2* mRNAs (Figure 3 A, C and D). These increases did not occur when PECs were exposed to TGF-β (5 ng/mL) combined with the anti-ENG antibody. TGF-β (5 ng/mL) had no effect on mRNA levels of *PDGFa*, *EGF*, *MCP1*, or *IL-6* expressed by PECs (Figure 3 B, E, F and G).

### Evaluation of chronic hypoxia-induced pulmonary hypertension in Eng^+/-^ and wild-type mice

Four groups of 8-week-old mice (8 *Eng^+/−^* and 5 *Eng^+/+^* mice) were established, with balanced numbers of males and females in each genotype group. The mice were exposed to chronic hypoxia (10% O_2_) or normoxia for 3 weeks. PAH was assessed based on RVSP, RV/(LV+S), and distal-artery muscularization after 3 weeks.

**Figure 4:**
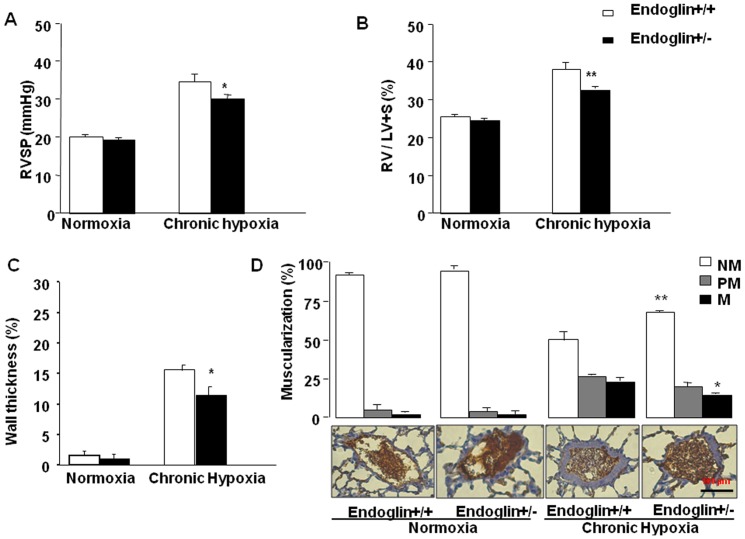
Mouse right ventricular (RV) systolic pressure (RVSP, mm Hg) (**A**). (**B**) RV hypertrophy reflected by the RV/(left ventricle+septum) weight ratio. (**C**) Percentage of lung vessel thickness. (**D**) Percentages of nonmuscularized (NM), partially muscularized (PM), and fully muscularized (M) lung vessels (top panel). Representative images for muscularization of distal pulmonary arteries (bottom panel). Values are mean±SEM. *P<0.05 and **P<0.01 compared with wild-type mice under the same conditions.

In normoxia, RVSP, RV/LV+S, and distal-artery muscularization were not significantly different between *Eng^+/−^* and wild-type mice (Figures 3A, B and C). After 3 weeks of chronic hypoxia, the increases in RVSP, RV/LV+S, and distal pulmonary artery muscularization were smaller in the *Eng^+/−^* mice than in the wild-type mice (Figure 4) (*P*<0.05).

### Evaluation of vascularization and inflammation in Eng^+/−^ and wild-type mice exposed to 3 weeks of chronic hypoxia

**Figure 5:**
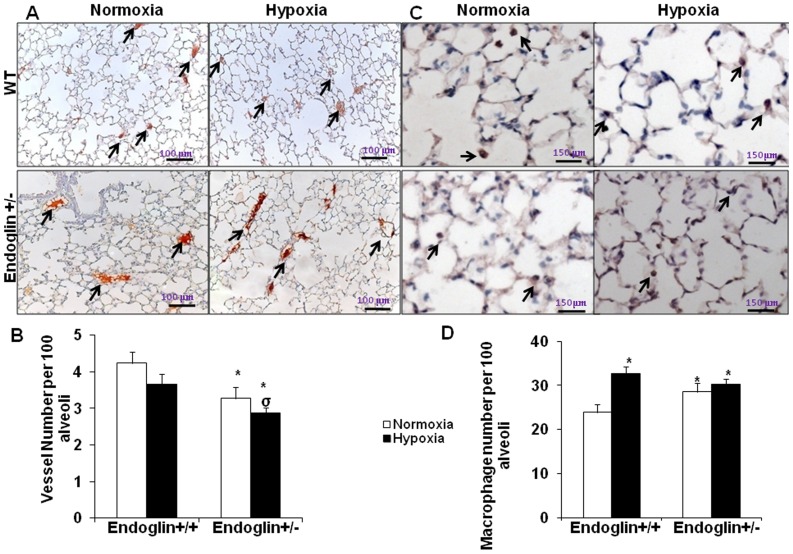
Mouse lung PECAM-1 immunostaining (**A**). (**B**) Number of vessels per 100 alveoli. (**C**) Mouse lung F4/80 immunostaining. (**D**) Number of macrophages per 100 alveoli. Values are mean±SEM. *P<0.02 compared with wild-type mice exposed to normoxia and ^σ^ *P*<0.02 compared with wild-type mice exposed to hypoxia.

In normoxia, vessel density was significantly lower in the *Eng^+/−^* mice compared to the wild-type mice (*P*<0.02). Exposure to chronic hypoxia for 3 weeks did not affect the pulmonary vessel number in the *Eng^+/−^* or wild-type mice (Figure 5A and B). In normoxia, pulmonary macrophage infiltrates were more marked in the *Eng^+/−^* mice than in the wild-type mice. Chronic hypoxia induced pulmonary macrophage infiltration in the wild-type mice but not in the *Eng^+/−^* mice (Figure 5C and D).

### Growth factors and Cytokine profile induced by chronic hypoxia in Eng^+/−^ and wild-type mice

**Figure 6:**
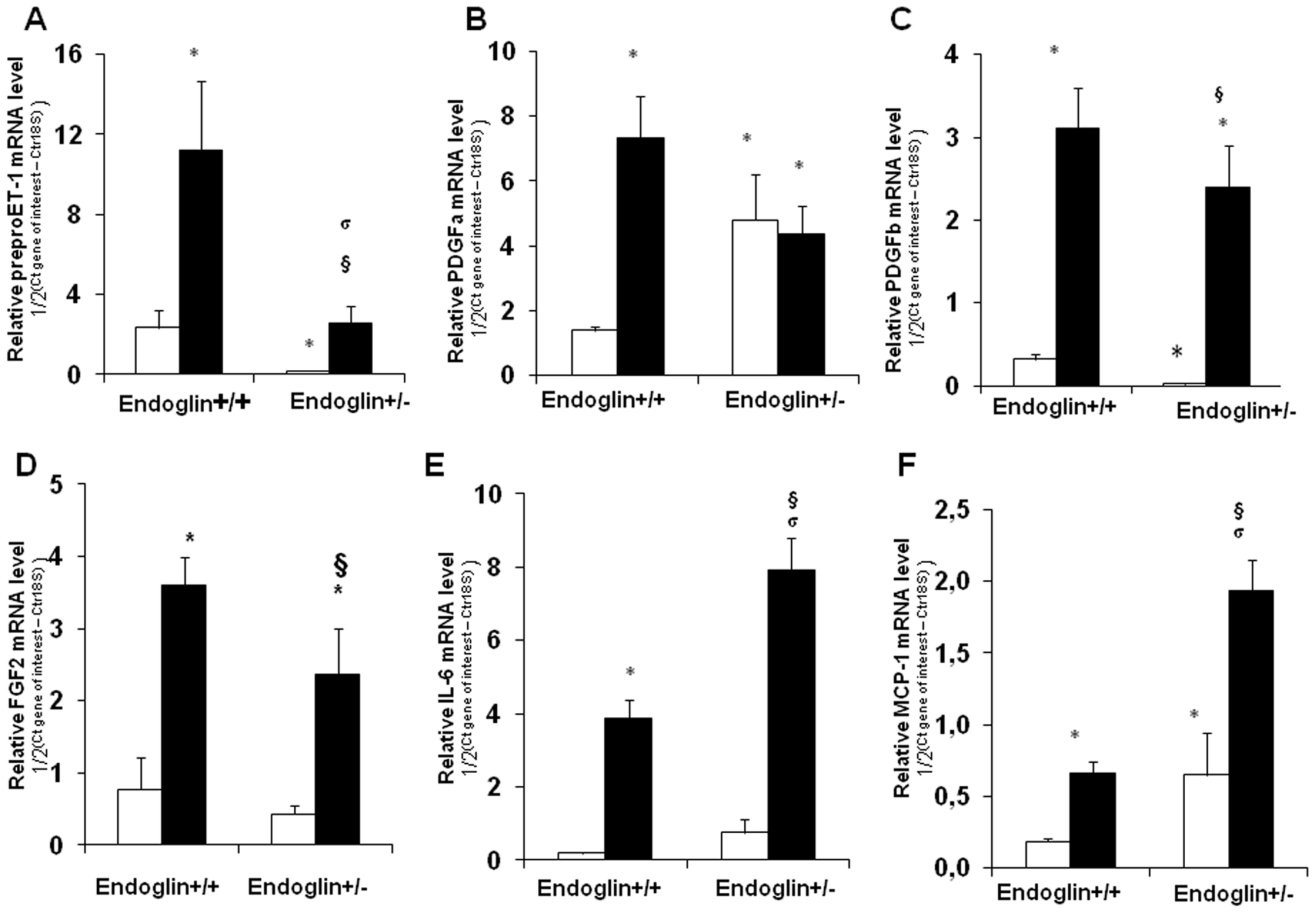
Mouse lung relative mRNA levels of (A) *preproET-1*, (B) *PDGFa*, (C) *PDGFb*, (D) *FGF2*, (E) *IL-6*, and (F) *MCP-1* in normoxia (open bars) versus hypoxia (closed bars). Values are mean±SEM.*P<0.05 compared to wild-type mice exposed to normoxia, ^§^ *P*<0.05 compared to *Eng^+/−^* mice exposed to normoxia, and ^σ^ *P*<0.05 compared to wild-type mice exposed to hypoxia.

We evaluated the expression of genes coding for ET-1, growth factors, and inflammatory mediators previously shown to play a key role in PAH progression and pulmonary arteriole remodeling. Chronic hypoxia (10% O_2_) induced an increase in mRNA levels of *preproET-1*, which remained lower in *Eng^+/−^* than in wild-type mice. Similarly, *PDGFb* and FGF2 induction in response to chronic hypoxia was less marked in lungs from *Eng^+/−^* mice than from wild-type animals. In contrast, the increase in MCP-1 and IL-6 mRNA levels under hypoxia was more marked in *Eng^+/−^* mice than in the wild-type mice, whereas no significant differences occurred for *PDGFa* (Figure 6B–G).

## Discussion

The main findings from this study are as follows: (i) compared to control specimens, lung tissue and PECs from patients with iPAH expressed increased amounts of ALK1 and ENG located predominantly on endothelial cells; (ii) compared to controls, patients with iPAH had higher serum and lung TGF-β levels; (iii) PECs from patients with iPAH exhibited Smad1/5/8 phosphorylation in response to increasing doses of TGF-β; (iv) medium from PECs treated with TGF-β markedly increased PA-SMC growth, and this effect seemed related to induction by TGF-β of *ET-1*, *PDGFb*, and *FGF2* expression in PECs and disappeared in the presence of anti-ENG antibody; and (v) ENG-deficient mice were partly protected against chronic hypoxia-induced PAH to wild-type mice, a finding that seemed related to decreased expression of PDGFa, PDGFb, and FGF2, three factors playing a key role in vascular remodeling and in the development of human and experimental PAH.

*ALK1* and *ENG* mutations have been associated with HHT and, to a lesser extent, heritable PAH [2], two familial vascular dysplasias with apparently opposite phenotypes. Thus, HHT is characterized by dilated vessels, telangiectasia, and arteriovenous malformations in the lung, liver, and brain. In the lungs, the arteriovenous malformations can result in right-to-left shunts, leading to severe cyanosis and dyspnea, and potentially to the development of pulmonary vascular remodeling with PAH. Various physiological factors, such as blood flow (shear stress) or pressure (cyclic strain), have been shown to trigger the vessel remodeling process, which involves PA-SMC proliferation and extracellular matrix protein synthesis and accumulation. Taken in concert, these data highlight the importance of the TGF-β/ALK1/ENG signaling pathway in maintaining vascular integrity.

Increased expression of TGF-β and its receptors ALK1 and ENG led to an increase in TGF-β/ALK1/ENG signaling activity in lung tissue and PECs from iPAH patients. Several studies have assessed the contribution of TGF-β to PAH, which remains debated. A recent study found decreased pulmonary TGF-β mRNA expression in PAH patients [9], contrasting with increases in TGF-β1 [10] or TGF-β isoforms 2 and 3 in previous studies [11]. These discrepancies may be ascribable to differences in measurement techniques: the previous studies relied on mRNA analysis [9] or TGF-β protein measurement in pulmonary arteries [10], [11], whereas we measured both lung and serum TGF-β protein contents. Upregulation of TGF-β has also been reported in several animal models of PAH [12], [13], and decreased TGF-β signaling related to dominant negative TGF-β type II receptor (TGF-βRII) overexpression [12], [14] or anti-TGF-β antibody [15] protects against PAH. Over the last 10 years, the importance of ALK1 and ENG in the pathogenesis of PAH has been established, notably by the identification of gene mutations [2], [16]. More recently, a nonsense mutation of Smad8, a component of the TGF-β/ALK1/ENG signaling pathway, was described in a patient with iPAH [17]. Data on the role for the TGF/ALK1 pathway in experimental models are conflicting. Thus, ALK1 was upregulated in monocrotaline-induced PAH [18], TGF-β and ALK1 where increased in a lamb model of congenital heart disease [19], whereas ALK1 and ENG were downregulated in another study [20]. Although it has been shown that Eng+/− and Alk1+/− mice spontaneously develop pulmonary hypertension based on the age [9]. Here, we found ALK1 and ENG overexpression in lungs from iPAH patients. In keeping with an earlier study [9], we found no significant change in *ALK5* mRNA expression in lungs from iPAH patients. In pulmonary arteries, PECs seem to be the main target of TGF-β, notably in iPAH. Thus, PECs from iPAH patients exhibited ALK1 and ENG overexpression without changes in ALK5 expression.

ENG was previously shown to play a pivotal role not only in the balance between TGF-β/ALK1 and TGF-β/ALK5 signaling [21], but also in determining the endothelial-cell growth potential. Here, we showed that PEC medium stimulated PA-SMC growth and that this effect was more marked when the PECs were previously incubated with low-dose TGF-β. These results support our previous finding that cultured PECs constitutively produce and release growth-promoting factors that act on PA-SMCs [5]. We also showed that PEC treatment with TGF-β induced overexpression of *ET-1*, *PDGFb*, and *FGF2*, three factors that may play a key role in PA-SMC growth and PAH development [22]–[24]. There are several reports that ALK1 activation by TGF-β or by bone morphogenetic protein (BMP)-9 induces the production of the vasoconstricting and mitogenic compound endothelin (ET)-1 [25], [26]. Recent studies also indicate that TGF-β induces endothelial IL-6 secretion [27], myofibroblast proliferation [28], and leukocyte migration [29], which are involved in the pathogenesis of PAH [23]. On the other hand, TGF-β-treated PECs from patients with iPAH showed a more sensitive activation of the Smad1/5/8 and, to a lesser degree, Smad2/3 signaling pathways, suggesting a predominance of the TGF-β/ALK1/Smad1/5/8 signaling pathways in these cells.

In the second part of our study, we investigated the consequences of ENG deficiency on PH development in mice. Because *Eng^−/−^* mice die during embryogenesis due to defects in vascular and cardiac maturation [7], we used engineered *Eng^+/−^* mice, previously described as an experimental model of HHT [7]. ENG deficiency in our *Eng^+/−^* mice partly prevented the development of chronic hypoxia-induced PH, as assessed based on RVSP, RV/(LV+S), and pulmonary arteriolar remodeling, compared with wild-type mice. Seeing that endoglin is determinant in the control of vascular density and that vessel numbers were inversely related to the severity of pulmonary hypertension, here we tried to establish a link between the degree of PH and vascular density in mice after exposure to chronic hypoxia. Our results show that, despite the reduction of the pulmonary vascular density, the *Eng^+/−^* mice were protected against the development of chronic hypoxic PH. Ardelean et al reported decrease of lung microvascular density and right ventricular hypertrophy in *Eng^+/−^* mice [30]. The discrepancy between the results could be due to background or age difference. In addition, *Eng^+/−^* mice exhibited increased pulmonary macrophage infiltration. This inflammation has been described as a potential precipitant of vascular bleeding in HHT [7] but may also be important in the pathogenesis of PAH [22]. However, *Eng^+/−^* mice were partly protected against hypoxia-induced PH compared to wild-type mice, suggesting that this underlying inflammatory phenotype did not exacerbate the effects of chronic hypoxia. Nevertheless, IL-6 and MCP-1, two cytokines previously implicated in PAH [22], were expressed at higher levels, both in normoxic and in hypoxic *Eng^+/−^* mice. The higher expression levels of these cytokines were in accordance with the degree of macrophage infiltration, which was more marked in lungs from *Eng^+/−^* mice.

We investigated the mechanism by which *Eng^+/−^* mice were partially protected against hypoxic PH and we measured growth factors involved in the development of human and experimental PAH. In keeping with previous data on PAH [23], chronic hypoxia was associated with increased *preproET-1* expression in both genotypes but the increase was significantly smaller in *Eng^+/−^* mice than in wild-type mice.

Because PA-SMC hyperplasia is among the main pathological changes in patients with PAH, we focused on the consequence of ENG deficiency in growth factor production in our experimental PAH model. Chronic hypoxia exposure was followed by increases in lung levels of PDGFb and FGF2 mRNA, but the levels of both growth factors remained significantly lower in *Eng^+/−^* mice than in wild-type mice. These *in vivo* results were consistent with the *in vitro* findings obtained using human PECs. These last data suggest that the protective effect against chronic hypoxic PH may be related to alterations in the PDGFb and FGF2 pathways. Indeed, previous studies showed that both growth factors played a key role in human and experimental PAH. The production of these growth factors or their receptors is increased in human PAH [22], [24], [31], [32]. Furthermore, inhibiting PDGFb or FGF2 synthesis using SiRNA or receptor antagonists protects and/or reverses PAH in experimental models.

Our study establishes a key role for the TGF-β/ALK1/ENG signaling pathway in PAH and suggests that TGF-β may act upstream to pathways that are crucial in PAH in both humans and rodents, such as the Endothelin1, PDGFb, and FGF2 pathways.

## Supporting Information

Table S1 (DOCX)
