# Supplementary material for: Key Role of the Endothelial TGF-β/ALK1/Endoglin Signaling Pathway in Humans and Rodents Pulmonary Hypertension
Source: PLoS One. 2014 Jun 23;9(6):e100310. doi: 10.1371/journal.pone.0100310 (PMC4067299; doi:10.1371/journal.pone.0100310)
Supplement: Table S1 — (DOCX) [file pone.0100310.s001.docx]

**Table S1**

|  | **Primer sequences (5′–3′)** |  | **Primer sequences (5′–3′)** |
| --- | --- | --- | --- |
| **Gene** | **Forward and Reverse** | **Gene** | **Forward and Reverse** |
| Human FGF2 | ACGGCGTCCGGGAGAA | Murine FGF2 | CCAACCGGTACCTTGCTATGA |
|  | ACACTCCCTTGATGGACACAACT |  | TTCCGTGACCGGTAAGTATTGTAG |
| Human EGF | ATGGTGGCGTGTGCATGTAT | Murine EGF | CCAAACGCCGAAGACTTATCC |
|  | ATCGTTCTCCAATATAGCCAATGAC |  | CTTATTACCGATGGGATAGCCC |
| Human PDGF-a | TTTTCTGCCATGCCTAAGTGTG | Murine PDGF-a | GGTCCACCACCGCAGTGT |
|  | GTGGAAAGTCATTCATCACAGGG |  | GGACCTCTTTCAATTTTGGCTTC |
| Human PDGF-b | GCACACGCATGACAAGACGGC | Murine PDGF-b | CTGAGGAACTGTATGAAATGCT |
|  | AGGCAGGCTATGCTGAGAGGTCC |  | CCATCTTCATCTACGGAGTCTC |
| Human ET-1 | TATCAGCAGTTAGTGAGAGG | Murine ET-1 | TGTGTCTACTTCTGCCACCT |
|  | CGAAGGTCTGTCACCAATGTGC |  | CACCAGCTGCTGATAGATAC |
| Human MCP-1 | GATCTCAGTGCAGAGGCTCG | Murine MCP-1 | TGATCCCAATGAGTAGGCTGGAG |
|  | TGCTTGTCCAGGTGGTCCAT |  | ATGTCTGGACCCATTCCTTCTTG |
| Human IL-6 | AATTCGGTACATCCTCGACGG | Murine IL-6 | GAGGATACCACTCCCAACAGACC |
|  | GGTTGTTTTCTGCCAGTGCC |  | AAGTGCATCATCGTTGTTCATACA |
| Human TGF-β_1_ | TCCTGCTTCTCATGGCCA |  |  |
|  | CCTCAGCTGCACTTGTAG |  |  |
| Human Alk1 | GTCCTGGTTCCGGGAGA |  |  |
|  | TTGCTCTTGACCAGCACA |  |  |
| Human Alk5 | GACATCCCTGGTCCATCC |  |  |
|  | TCTGGCACAACTCCACTG |  |  |
| Human Endoglin | GCATCCTTCGTGGAGCTA |  |  |
|  | GACACCTGCATGCCACA |  |  |
